# Non-traumatic complications of a solitary rib osteochondroma; an unusual cause of hemoptysis and pneumothorax

**DOI:** 10.1259/bjrcr.20200015

**Published:** 2020-03-20

**Authors:** VE Versteegh, WN Welvaart, EEM Oberink-Gustafsson, A Lindenholz, GHA Staaks, CM Schaefer-Prokop

**Affiliations:** 1 Department of Radiology and Nuclear Medicine, Meander MC, Amersfoort, The Netherlands; 2 Department of Surgery, Meander MC, Amersfoort, The Netherlands; 3 Department of Pediatrics, Meander MC, Amersfoort, The Netherlands; 4 Department of Pulmonology, Meander MC, Amersfoort, The Netherlands

## Abstract

Osteochondromas are a very common and usually asymptomatic entity which may originate anywhere in the appendicular and axial skeleton. However, the ribs are a rare site of origin and here they may prove symptomatic for mechanical reasons. In this case report, we describe an unusual case of a symptomatic osteochondroma of the rib secondary to its location and unique shape, ultimately requiring surgical intervention.

## Introduction

Osteochondromas, also known as exostoses, are common benign developmental osteochondromatous proliferations. They are estimated to occur in 3% of the general population and account for 20–50% of all benign bone tumors and 10–15% of all bone tumors.^1–5^

Mostly they develop as a solitary bone proliferation, however up to 15% of osteochondromas occur in the spectrum of Hereditary Multiple Exostoses syndrome.^1^

Osteochondromas can originate anywhere in the appendicular or axial skeleton, however the majority develops in the lower limb with a prevalence of 50% around the knee.^4^

Although osteochondromas are benign in nature, they may cause symptoms for mechanical reasons and rarely may undergo malignant transformation.^1–5^

In this case report, we present a rare case of a solitary osteochondroma of the rib causing significant pulmonary symptoms due its unique shape and location.

## Case report

A healthy 15-year-old boy presented to the Emergency Department with a 2-week history of a persistent productive cough. The sputum occasionally contained fresh blood in small quantities. He had been on a short haul flight a few days prior to symptom onset.

There was no history of fever. Family history was positive for ankylosing spondylitis only.

He had recently seen the general practitioner, who had prescribed Codeine with limited effect.

At presentation he was hemodynamically stable. Physical examination revealed a left-sided pleuritic rub only. Blood investigations demonstrated a Hb of 8.4 mmol l^−1^ with normal clotting and infection parameters.

**Figure 1.**
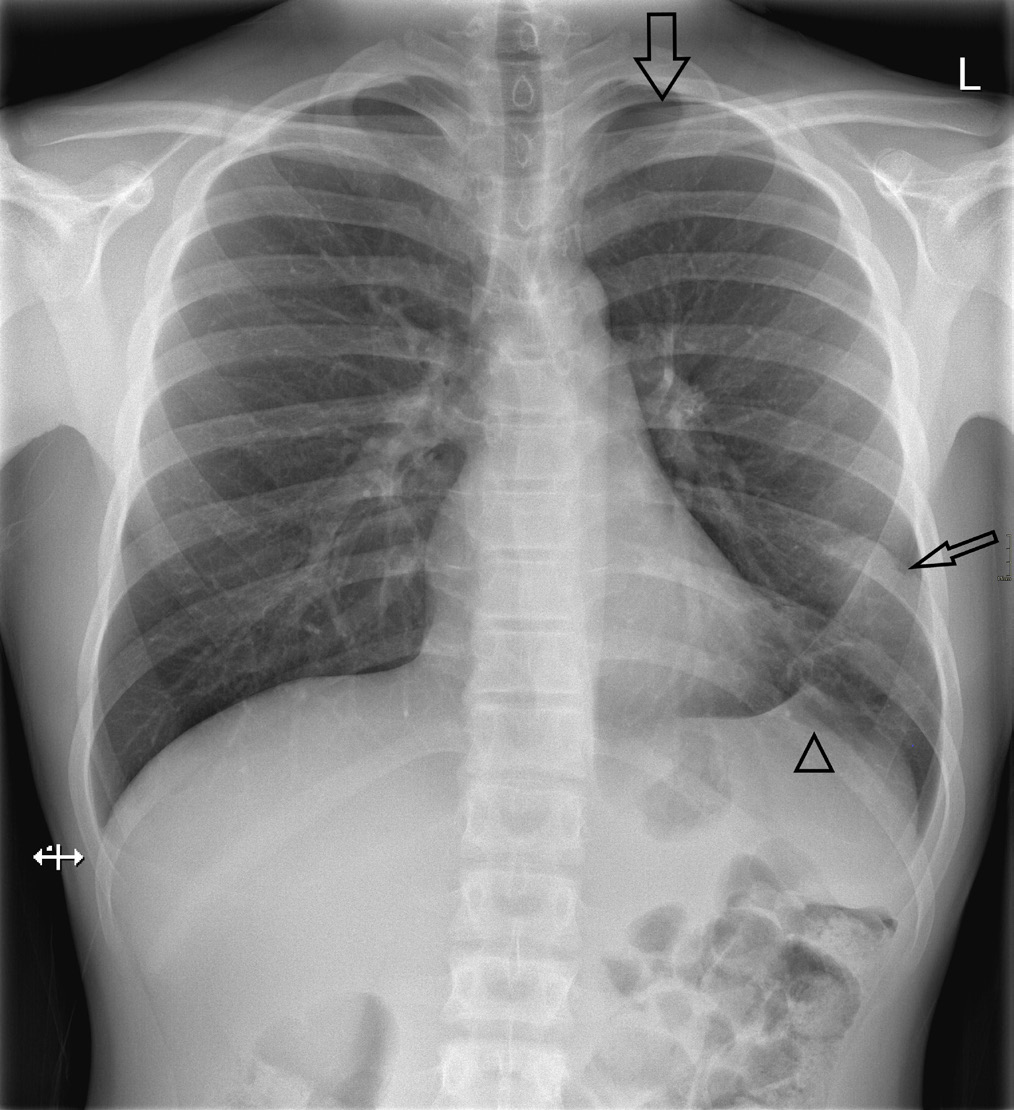
X-Thorax showing a leftsided apical pneumothorax (large arrow), left upper lobe consolidation (small arrow) and basal pleural adhesion (arrow head).

A chest radiograph was performed and showed a small left-sided apical pneumothorax with a basal pleural adhesion and consolidation in the basal left upper lobe (Figure 1).

Subsequently, a CT angiography of the thorax was performed to rule out potential pulmonary embolisms and for further investigation of the radiograph findings.

**Figure 2.**
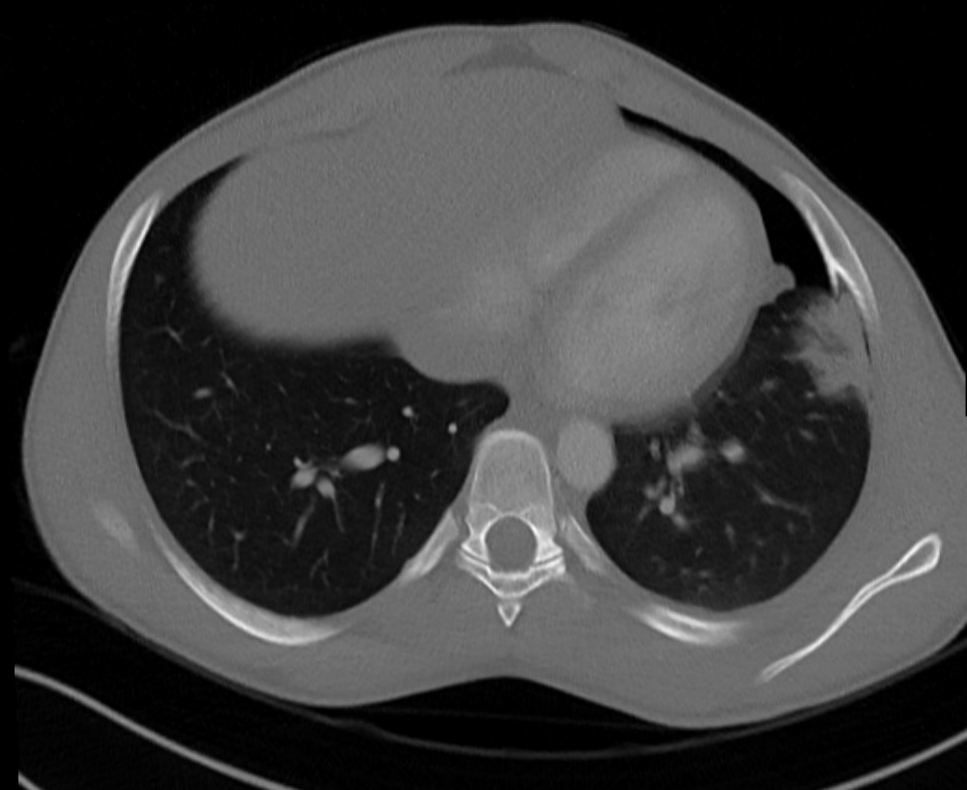
Axial CT angiography image (5 mm reconstruction; bone setting) showing osseous continuity of the osteochondroma and the sixth rib on the left side.

**Figure 3.**
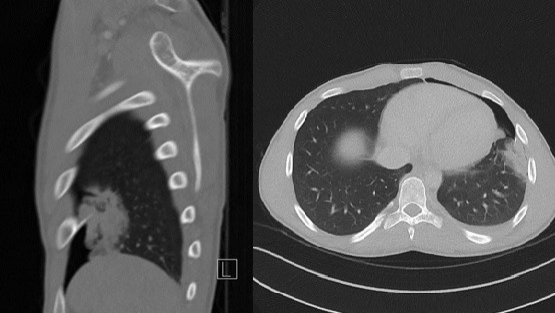
Sagittal and axial reconstruction CT angiography (in bone and pulmonary setting respectively), showing an osseous proliferation perforating the lung parenchyma causing a pneumothorax and pulmonary hemorrhage.

**Figure 4.**
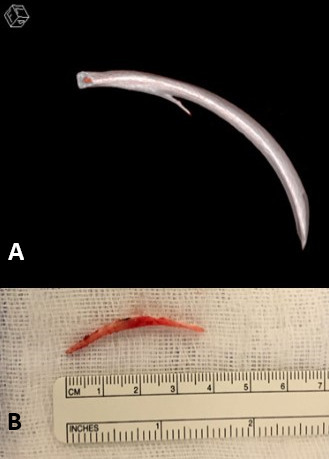
(a) 3D volume rendering reconstruction of CT angiography (Syngo.via, Siemens (Erlangen; Germany)) showing the osteochondroma in our patient originating from the 6th rib and its continuity with the underlying bone. (b) Post-operative specimen after resection of the osteochondroma. 3D, three-dimensional.

No signs of pulmonary embolism were seen. Instead, there was a left-sided pneumothorax, associated with a consolidation in the lingula and basal pleural adhesion. The underlying reason for these findings was a 4 cm long spear-shaped osseous proliferation originating from the visceral side of the left ventrolateral sixth rib (Figures 2 and 3). There was no visible extravasation of intravenous contrast. The osseous proliferation showed medullary continuity with the parent rib-bone which prompted the diagnosis of osteochondroma perforating the lung parenchyma and causing an air-leak, pulmonary contusion and intraparenchymal hematoma (Figures 2–4).

**Figure 5.**
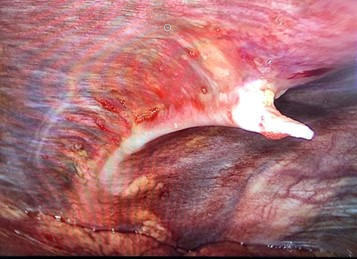
Per-operative view of our patient illustrating the osteochondroma compromising the thoracic cavity.

**Figure 6.**
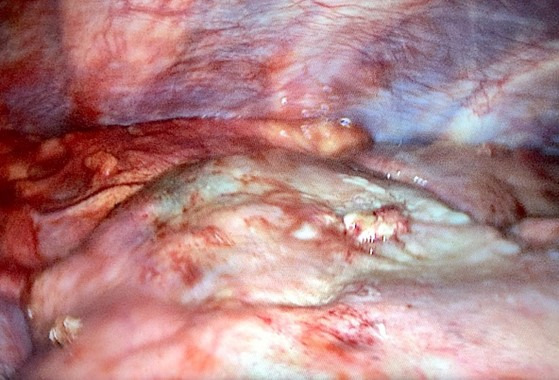
Per-operative view of our patient illustrating the pulmonary hemorrhage adjacent to the osteochondroma.

The patient underwent an uniportal video assisted thoracic surgery the following day confirming the CT findings of an internally directed sharp osseous proliferation originating from the sixth rib perforating the visceral pleura (Figure 5). Adjacent to the osseous proliferation a local pulmonary hemorrhage of the upper lobe was seen (Figure 6). The osseous proliferation was subsequently resected and diathermic coagulation of the pleura was performed to prevent air leakage and recurrence.

A post-operative chest radiograph showed complete resorption of the pneumothorax and the patient was discharged after 2 days in good clinical condition.

Histological examination confirmed the osseous proliferation consistent with a osteochondromatous proliferation with no signs of dysplasia or concern of malignancy. Pleural specimens showed reactive and old hemorrhagic changes.

## Discussion

This case is a rare example of a solitary osteochondroma leading to serious complications because of its shape and location.

Osteochondromas are considered to develop as a chondromatous epiphyseal herniation through the periosteal cuff which implant intracortically in the metaphysis. They tend to grow away from the joint through tensile forces of tendons and ligaments. Radiologically, they show medullary and cortical continuity with the parent bone and a small hyaline cartilage cap, generally smaller than 2 cm^2,4^.

Most osteochondromas are incidental findings, however they may cause symptoms ranging from deformity, fractures or mechanical problems with compromise of neurovascular bundle, impingement on ligaments or tendons or secondary bursae.^1,4^ These symptomatic osteochondromas usually present in younger patients, 75–80% are discovered before the 20th year of age.^4^

Primary chest wall tumors are very rare and account for 5–10% of bone tumors, 95% of which occur in the ribs^5^ and are mostly benign (55%).^3^ Fibrous dysplasia is the most common cause of a benign rib growth (22–30%),^3,5^ followed by osteochondromas (3–8%).^2,3^

It is estimated that 55% of rib osteochondromas occur in patients with Hereditary Multiple Exostoses.^6^

Osteochondromas of the ribs generally originate near the costochondral junction but may also occur at the costovertebral junction. Although our patient presented with an osteochondroma slightly more lateral to the costochondral junction, it was histopathologically confirmed to be consistent with a benign osteochondromatous proliferation.

When symptomatic, they can cause pulmonary symptoms such as pneumothorax,^7–10^ and hemothorax^7,11–13^ or simply a palpable lump.^14^

Cases have also been rarely reported with extrapulmonary symptoms ranging from acute coronary syndrome due to extrinsic compression,^15^ pericardial thickening,^16^ hiccups,^17^ diaphragmic erosion and rupture^7,18^ and spinal cord compression.^19^

No significant traumatic event prior to symptoms has been described in these case reports and neither was the case in our patient. However, some patients had a history of previous athletic activity^8,9,12^ and labor.^15^ The absence of a provoking event is consistent with a large population-based study which showed 76% of spontaneous pneumothorax in the general population occur in rest or sleep.^20^

Our patient reported cough and hemoptysis shortly after an intercontinental flight. Pulmonary embolism was therefore excluded. Pneumothorax and air travel do have an known association but this is considered to be the result of rupture of a superficial lung cyst due to volume expansion with drop in barometric pressure at higher altitude.^21^

Although there was no known history of underlying lung disease, we cannot rule out volume expansion of the lung as a predisposing factor for a traumatic pneumothorax in our patient.

## Conclusion

We presented a case of a solitary osteochondroma of the ventrolateral sixth rib causing a pneumothorax, pulmonary hemorrhage and hemoptysis due to its unique shape and location.

Osteochondromas are very common, however the ribs are a rare site of origin.

These osteochondromas tend to be asymptomatic, but may cause symptoms for mechanic or cosmetic reasons.

In the case of a patient with a symptomatic osteochondroma of the rib, symptoms may range from hemo- or pneumothorax to cardiac symptoms, diaphragmic rupture and spinal nerve injuries due to extrinsic compression.

## Learning points

- Osteochondromas are a very common entity, but rarely occur at the ribs.
- Osteochondroma of the rib may cause non-specific, but serious complications for mechanical reasons depending on its location.
- Surgical intervention should be considered in the case of a symptomatic osteochondroma of the rib.
